# Growth hormone reduces aneuploidy and improves oocytes quality by JAK2-MAPK3/1 pathway in aged mice

**DOI:** 10.1186/s12967-023-04296-z

**Published:** 2023-06-29

**Authors:** Yun-Yao Luo, Xi Zeng, Ling Zhu, Chong Li, Juan Xie, Qiang Dong, Qing-Yuan Sun, Guo-Ning Huang, Jing-Yu Li

**Affiliations:** 1 Chongqing Key Laboratory of Human Embryo Engineering, Center for Reproductive Medicine, Women and Children’s Hospital of Chongqing Medical University, Chongqing, 400010 China; 2 Chongqing Clinical Research Center for Reproductive Medicine, Chongqing Health Center for Women and Children, Chongqing, 400010 China; 3 Guangzhou Key Laboratory of Metabolic Diseases and Reproductive Health, Reproductive Medicine Center, Guangdong Second Provincial General Hospital, Guangzhou, 510310 China

**Keywords:** Aged oocytes, Growth hormone, Aneuploidy, Oocyte quality, MAPK3/1, JAK2

## Abstract

**Background:**

The global delay in women’s reproductive age has raised concerns about age-related infertility. The decline in oocyte quality is a limiting factor of female fertility, yet there are currently no strategies to preserve oocyte quality in aged women. Here, we investigated the effects of growth hormone (GH) supplementation on aneuploidy of aged oocytes.

**Methods:**

For the in vivo experiments, the aged mice (8-month-old) were intraperitoneally injected with GH daily for 8 weeks. For the in vitro experiments, germinal vesicle oocytes from aged mice were treated with GH during oocyte maturation. The impacts of GH on ovarian reserve before superovulation was evaluated. Oocytes were retrieved to assess oocyte quality, aneuploidy and developmental potential characteristics. Quantitative proteomics analysis was applied to investigate the potential targets of GH in aged oocytes.

**Results:**

In this study, we demonstrated that GH supplementation in vivo not only alleviated the decline in oocyte number caused by aging, but also improved the quality and developmental potential of aged oocytes. Strikingly, we discovered that GH supplementation reduced aneuploidy in aged oocytes. Mechanically, in addition to improving mitochondrial function, our proteomic analysis indicated that the MAPK3/1 pathway may be involved in the reduction in aneuploidy of aged oocytes, as confirmed both in vivo and in vitro. In addition, JAK2 may also act as a mediator in how GH regulates MAPK3/1.

**Conclusions:**

In conclusion, our research reveals that GH supplementation protects oocytes against aging-related aneuploidy and enhances the quality of aged oocytes, which has clinical significance for aged women undergoing assisted reproduction technology.

**Graphical Abstract:**

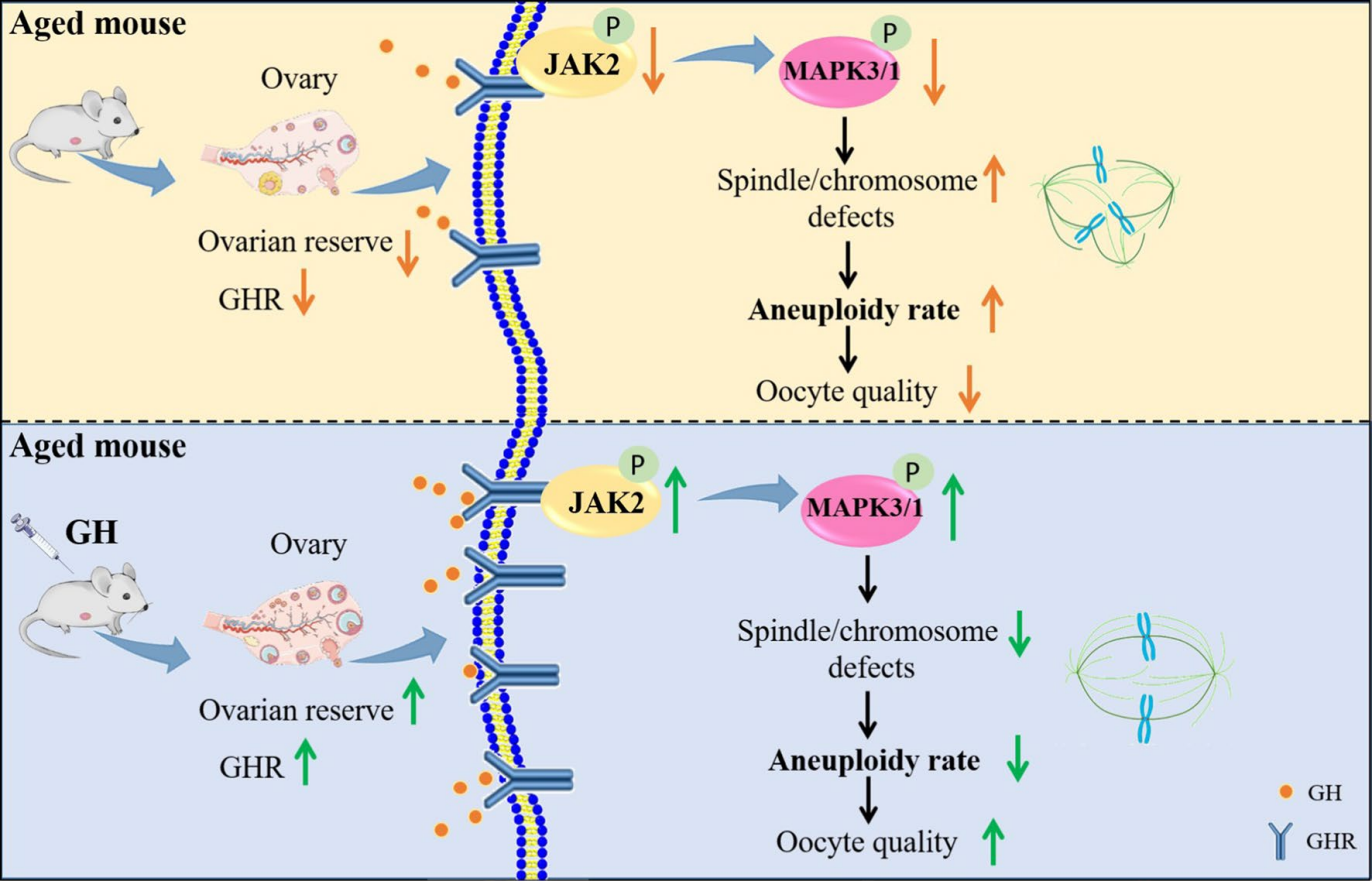

**Supplementary Information:**

The online version contains supplementary material available at 10.1186/s12967-023-04296-z.

## Introduction

The global trend of delayed childbearing has drawn attention to age-related infertility. The proportion of women having their first child after the age of 35 has increased tenfold since 1970, and the average age of first birth has climbed from 21 to 26.9 years over the last three decades [1, 2]. Female fertility is reduced by low-quality of oocytes brought on by aging. For instance, oocyte quality dramatically decreases after the age of 35 years, which causes a sharp increase in infertility [3, 4].

Oocyte aging is a decline in oocyte quality, including loss of chromosomal integrity, spindle defects, and low developmental potential in oocytes. Oocyte aging is caused by a number of biological processes, including mitochondrial dysfunction, an accumulation of reactive oxygen species (ROS), energy metabolism disorders, and epigenetic changes [5–7]. Aged oocytes produce less ATP, have fewer mitochondria, and exhibit disrupted mitochondrial function, all of which cause chromosomal displacement and spindle damage, ultimately leading to aneuploid oocytes. Aged oocytes have a high incidence of aneuploidy, which impairs their developmental potential and reduces the fertilization rate. This can result in pregnancy loss, embryo mortality, and congenital birth defects [8–10]. Currently, antioxidant like melatonin, coenzyme Q10, and α-lipoic acid are used to improve the quality of aged oocytes [11–13], however, the potential mechanisms remain unclear.

Growth hormone (GH), a peptide primarily secreted by the anterior pituitary gland, regulates growth, glucolipid metabolism, and immune functions via multiple mechanisms [14]. In addition, GH exerts an anti-aging effect by increasing bone density and decreasing the risk of cardiovascular disease [15, 16]. GH is also involved in the regulation of female reproduction. GH bound to GH receptor (GHR) augments the effects of gonadotropin on oocytes and granulosa cells, which may also enhance steroidogenesis, follicular development and oocyte maturation [14, 17]. In assisted reproductive technology (ART), GH is used as an adjunct to ovarian stimulation in patients with a poor ovarian response (POR), although its role is still controversial. Several studies have reported that GH improves ART outcomes in POR patients [18, 19], consistent with our recent reports that GH supplementation increases the clinical pregnancy and live birth rates [20, 21]. In animal models, GH supplementation has recently been shown to improve the oocyte quality by enhancing mitochondrial function [22, 23]. However, whether GH reduces the aneuploidy in aged oocytes, and if so the underlying mechanisms, are unclear.

In this study, we showed that GH supplementation in vivo reduced the incidence of aneuploidy, thereby increasing the quality and developmental potential of aged oocytes. We discovered through proteomic analysis that GH may lessen aneuploidy via JAK2- MAPK3/1 pathway in addition to enhancing mitochondrial functions.

## Materials and methods

### Animals

All procedures were performed in strict accordance with the 1988 guidelines of the State Scientific and Technological Commission of China for the use of laboratory animals. The protocols were approved by the Ethics Committee of the Chongqing Health Center for Women and Children. Young (8 weeks old) and aged (32 weeks old) female C57BL/6 J mice were purchased from SPF Biotechnology Company (Beijing, China) and maintained under controlled temperature (20–23 ℃) and illumination (12 h light/dark cycle) conditions with ad libitum access to water and food. After treatment, mice were sacrificed at 40 weeks old by cervical dislocation. Every effort was made to minimize suffering during the collection of oocytes.

### GH, CEP-33779, and U0126 treatment

For the in vivo experiments, the aged mice were randomly divided into two groups. Mice in one group were intraperitoneally injected with GH at 3 every afternoon (GenSci, Changchun, China; 1.6 mg/kg body weight) for 8 weeks; those in the other group were intraperitoneally injected with an equivalent volume of normal saline (NS). Young mice, as young controls, were also injected with NS. For the in vitro experiments, germinal vesicle (GV) oocytes from aged mice were treated with or without GH (100 ng/mL) in M16 medium. The JAK2 and MAPK3/1 inhibitors were diluted with phosphate-buffered saline (PBS). The JAK2 inhibitor CEP (#S2806; Selleckchem, Houston, TX) and MAPK3/1 inhibitor U0126 (#S1102; Selleckchem, Houston, TX) were administered in concentrations of 80 nM and 4 μM, respectively.

### Histological analysis of ovaries

Ovaries were fixed in 4% paraformaldehyde (PFA) overnight at 4℃ and sectioned at 6 μm thickness for H&E staining. To classify and count follicles, 20 slices were obtained from each sampled ovary. Preantral, antral, and atretic follicles were enumerated as described previously [24]*.* The number of follicles were enumerated by counting five random ovarian slides from three mice in each group. The average number of follicles on each slide was determined for statistics analysis.

### Oocyte collection and culture

Oocytes were prepared as described previously [25]*.* To collect fully-grown GV oocytes, female mice were injected with 10 IU of pregnant mare serum gonadotropin (PMSG; Sigma-Aldrich, St. Louis, MO). After 48 h, the cumulus-oocyte complex was obtained by artificially rupturing antral follicles. The GV oocytes were obtained after removal of cumulus cells by repeated mouth pipetting, and cultured in M16 medium (Sigma-Aldrich) with liquid mineral oil at 37 °C in an atmosphere of 5% CO_2_ for 12–14 h. Female mice were injected with 10 IU of human chorionic gonadotropin (hCG; Sigma-Aldrich) 48 h after PMSG initiation to collect ovulated mature oocytes. Oviducts were harvested 14–16 h after hCG administration, and the COCs released from the oviduct that were digested with hyaluronidase (Sigma-Aldrich).

### In vitro* fertilization (IVF) and embryo culture*

Spermatozoa were collected from adult male C57BL/6 J mice and capacitated for 1 h in HTF medium (Sigma-Aldrich). Metaphase II (MII) oocytes were incubated with sperm for 6 h in a humidified atmosphere of 5% CO_2_ at 37 ℃. The sperm concentration for fertilization was 1 × 10^6^/mL. Two-cell embryos were cultured in KSOM (Sigma-Aldrich), and the fertilization, cleavage, and blastocyst formation rates were calculated.

### Quantitative proteomics analysis

Peptides from 10 oocytes each (from three biological replication) from the GH + aged and aged groups were analyzed. The oocytes were sonicated three times on ice using a high-intensity ultrasonic processor (Scientz, Ningbo, China) in lysis buffer (8 M urea, 1% protease inhibitor cocktail). The protein concentration was measured by the Bradford method. Lysine crotonylation (Kcro), succinylation (Ksucc) and malonylation (Kmal) peptides were enriched using pre-washed antibody beads (PTM Biolabs, Hangzhou, China). The eluted fractions were combined and vacuum-dried. For liquid chromatography–tandem mass spectrometry (LC–MS/MS), the peptides were desalted with C18 ZipTips (Millipore, Billerica, MA) according to the manufacturer’s instructions. The resulting MS/MS data were processed using the MaxQuant search engine (v. 1.6.15.0). Tandem mass spectra were searched against the mice SwissProt database (20,422 entries) and a reverse decoy database. The false discovery rate (FDR) was adjusted to < 1%.

### Bioinformatics analysis

Differentially expressed (DE) proteins were identified using the *t*-test by python (Version: 2.7.17). A p-value < 0.05 and fold change > 1.5 were considered indicative of significance. Gene Ontology (GO) and Kyoto Encyclopedia of Genes and Genomes (KEGG) pathway enrichment analyses were performed using Metascape (Version: 3.5) and DAVID (Version: DAVID 2021) [26]. A p-value < 0.05 was considered indicative of significant enrichment. STRING was used to evaluate the predicted associations of DE proteins [27].

### Immunofluorescence and confocal microscopy

Oocytes were fixed in 4% PFA for 30 min and permeabilized in 1% Triton X-100 (Sigma) for 15 min at room temperature. Next, oocytes were blocked with 3% bovine serum albumin (BSA) for 1 h and incubated with anti-α-tubulin-FITC (fluorescein isothiocyanate, 1:500; #F2168; Sigma-Aldrich), anti-CREST (1:200; #CA95617; Antibodies Incorporated, Davis, CA), anti-GHR (1:50; #AF1360-SP; R&D Systems, Minneapolis, MN), anti-phospho-JAK2 (1:200; ab32101; Abcam, Cambridge, UK), and anti-phospho- MAPK3/1 (1:200; #4370 T; Cell Signaling Technology) antibodies at 4 ℃ overnight. After washing three times with PBS, oocytes were incubated with the corresponding secondary antibody at room temperature for 1 h. Oocytes were counterstained with an anti-Hoechst 33342 (1:1,000; #C1022; Beyotime, Shanghai, China) antibody for 15 min. Finally, oocytes were examined using a laser-scanning confocal microscope (TCS SP8; Leica, Wetzlar, Germany). Fluorescence intensity was analyzed using ImageJ software (NIH, Bethesda, MD).

For active mitochondria staining, oocytes were cultured in M2 medium (Sigma-Aldrich) containing anti-JC-1 (1:500; #C2005; Beyotime), anti-MitoTracker Red (1:500; #C1049; Beyotime), for 30 min in darkness at 37 ℃ and 5% CO_2_. After washing three times with fresh M2 medium (Sigma-Aldrich) for 10 min each, fluorescence intensity was measured as described previously.

### Chromosome spreading

MII-stage oocytes were placed in 5‰ HCl to remove the zona pellucida. When the zona pellucida appeared to be fully removed, oocytes were quickly transferred to M2 medium for 3 sequential washes. Then, oocytes were placed in hypotonic solution (1% KCl) at room temperature for 3–5 min to loosen chromosomes. Oocytes were transferred to slides and fixed in 1% PFA supplemented with 0.15% Triton X-100 and 3 mM dithiothreitol (Sigma). The slides were incubated in a moist chamber at room temperature for 6–8 h. After blocking with 3% BSA for 1 h, chromosomes were stained with CREST (1:200) and Hoechst 33,342 (1:1000) at 4°℃ overnight and counted under a confocal laser-scanning microscope.

### Sperm binding assay

Spermatozoa were collected from C57BL/6J male mice and capacitated for 1 h in HTF medium (Sigma-Aldrich). MII stage oocytes were incubated with sperm at 37 °C for 2 h and fixed in 4% PFA for 30 min. After staining with Hoechst 33,342 (1:1000) for 15 min, bound spermatozoa were enumerated under a confocal microscope.

### Quantitative real-time PCR

Total RNA from oocytes was extracted using the Arcturus PicoPure RNA Isolation Kit (Thermo Fisher, Waltham, MA), according to the manufacturer’s protocol. Extracted RNA was subjected to reverse transcription using PrimeScript RT Master Mix (TaKaRa, Dalian, China), and to quantitative real-time PCR using SYBR Green Mix (TaKaRa) and the primers listed in Additional file 1: Table S1. We used *Hprt* as the internal control to quantify gene expression. Experiments were performed in triplicate.

### WGA and DNA sequencing of oocytes

In this part, the oocytes of young (n = 26), aged (n = 26), GH + aged groups (n = 30) were used for DNA sequencing (from three biological replication). The MALBAC single-cell WGA method was used to amplify DNA from oocytes (the first polar body removed), following the manufacturer’s protocol (TaKaRa Bio USA, Inc., San Jose, CA). The DNA concentration was measured by Qubit^®^ DNA kit in Qubit^®^ 3.0 Flurometer (Invitrogen, USA). A total amount of 0.2 μg DNA per sample was used as input material for the DNA library preparations. Sequencing library was generated using NEB Next^®^ Ultra™ DNA Library Prep Kit for Illumina (NEB, USA) following manufacturer’s recommendations and index codes were added to each sample. Briefly, genomic DNA sample was fragmented by sonication to a size of 350 bp. Then DNA fragments were endpolished, A-tailed, and ligated with the full-length adapter for Illumina sequencing, followed by further PCR amplification. After PCR products were purified by AMPure XP system (Beckman Coulter, Beverly, USA), DNA concentration was measured by Qubit^®^3.0 Flurometer (Invitrogen, USA), libraries were analyzed for size distribution by NGS3K/Caliper and quantified by real-time PCR (3 nM). The clustering of the index-coded samples was performed on a cBot Cluster Generation System using Illumina PE Cluster Kit (Illumina, USA) according to the manufacturer’s instructions. After cluster generation, the DNA libraries were sequenced on Illumina platform and 150 bp paired-end reads were generated.

The original fluorescence image files obtained from Illumina platform are transformed to short reads (Raw data) by base calling and these short reads are recorded in FASTQ format, which contains sequence information and corresponding sequencing quality information. Sequence artifacts, including reads containing adapter contamination, low-quality nucleotides and unrecognizable nucleotide (N), undoubtedly set the barrier for the subsequent reliable bioinformatics analysis. Hence quality control is an essential step and applied to guarantee the meaningful downstream analysis. We used Fastp (version 0.19.7) to perform basic statistics on the quality of the raw reads.

### Time-lapse monitoring of embryo morphokinetics

Embryo developmental kinetics was evaluated using the EmbryoScope™ time-lapse imaging system (Vitrolife AB, Kungsbacka, Sweden). Embryos were cultured individually in 25 μL of medium, and delayed images were generated at 15-min intervals. Morphokinetic events were recorded as hours post-two pronuclei (2PN) fading (syngamy). Time t2 is the interval from syngamy (2PN fading) to cleavage to the2-cell stage. Times t3, t4, t5, t8, tM, tB are the times of division into 3-, 4-,5-, and 8-cell embryos, morula, and blastocysts, respectively [28].

### Oocyte live imaging

For live imaging, mRNA encoding H2B-Cheery (200 ng/μL) was microinjected into GV-stage aged oocytes. After incubation in milrinone for 2 h, images of live oocytes were acquired using a confocal laser-scanning microscope at 10 min intervals for 16 h.

### Statistical analysis

Values are means ± standard error of the mean (SEM) from at least three independent experiments, and were analyzed using the paired-samples *t*-test in Prism 7.0 software (GraphPad Software Inc., San Diego, CA). Significance was accepted at p < 0.05.

## Results

### GH supplementation improved the oocyte quality of aged mice

**Fig. 1:**
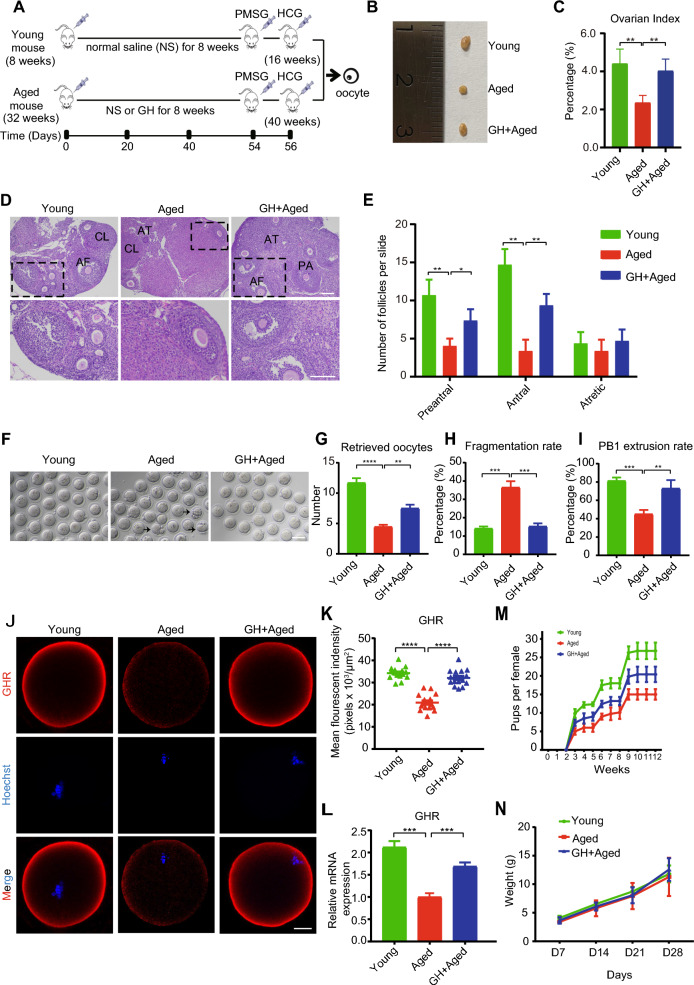
Effect of GH supplementation on ovary aging and the quality of aged oocytes. **A** Schedule of GH treatment and hormone administration to induce superovulation. **B ** and **C** Morphological features of ovaries. Ovarian index = (ovary weight/body weight) × 100. **D** H&E staining of ovarian sections from young, aged, and GH + aged mice. PA, preantral follicle; AF, antral follicle; AT, atretic follicle; CL, corpus luteum. The bottom panel is the enlarged view of the box area in the top panel. Scale bar, 200 μm. **E** Numbers of follicles at the indicated stages in young (n = 3), aged (n = 3), and GH + aged (n = 3) mice. **F** Representative images of in vivo matured oocytes from young, aged, and GH + aged mice. Black arrows, oocytes with fragmentation. Scale bar, 80 μm. **G** Numbers of oocytes after superovulation in young (n = 10), aged (n = 10), and GH + aged (n = 10) mice. **H ** and **I** Fragmentation and first polar body (PB1) extrusion rates in young (n = 39), aged (n = 35), and GH + aged (n = 42) oocytes. **J** Representative images of GHR in young, aged, and GH + aged oocytes. Scale bar, 20 μm. **K** and **L** Protein and mRNA levels of GHR revealed by immunofluorescence (n = 15) and real-time PCR (n = 25), respectively. **M** Cumulative number of pups over 12 weeks as a measure of the fertility of young (n = 6), aged (n = 6), and GH + aged (n = 6) mice. **N** Body weights of newborns (n = 24, 10, and 12 in the young, aged, and GH + aged groups, respectively) over 4 weeks. Data are means ± SEM of at least three independent experiments. *p < 0.05, **p < 0.01, ***p < 0.001, ****p < 0.0001

We explored the effect of GH supplementation on the aging-related reduction in the ovarian reserve. For this purpose, young (8-weeks-old) and aged mice (32-weeks-old) were intraperitoneally injected with GH or normal saline (NS) for 8 weeks, and superovulation hormone was administered on the 54th and 56th days (Fig. 1A). After treatment, the aged mice (40 weeks old) had smaller ovaries and a lower ovarian index than the young mice, and GH supplementation alleviated the effects of ovarian aging (Fig. 1B and C). H&E staining showed that the number of follicles at various developmental stages significantly decreased in aged mice. GH supplementation increased the numbers of preantral and antral follicles, but not atretic follicles (Fig. 1D and E). The number of ovulated oocytes and proportion of mature oocytes were significantly reduced in aged compared with young mice, whereas the rate of fragmentation increased (Fig. 1F–I). In contrast, GH supplementation improved the aging-related decline in the number, maturation, and morphology of oocytes (Fig. 1F–I). In addition, GH supplementation increased the protein level of GHR on the membrane of aged oocytes, as well as the mRNA level (Fig. 1J–L). Consistent with the increased number of oocytes, the fertility of aged mice was also improved by GH supplementation (Fig. 1M), while the body weight of newborns was comparable among the three groups (Fig. 1N). These results indicated that exogenous administration of GH increased the number and improved morphology of aged oocytes, and slightly increased the fertility of aged mice.

### GH supplementation restored mitochondrial function in aged oocytes

**Fig. 2:**
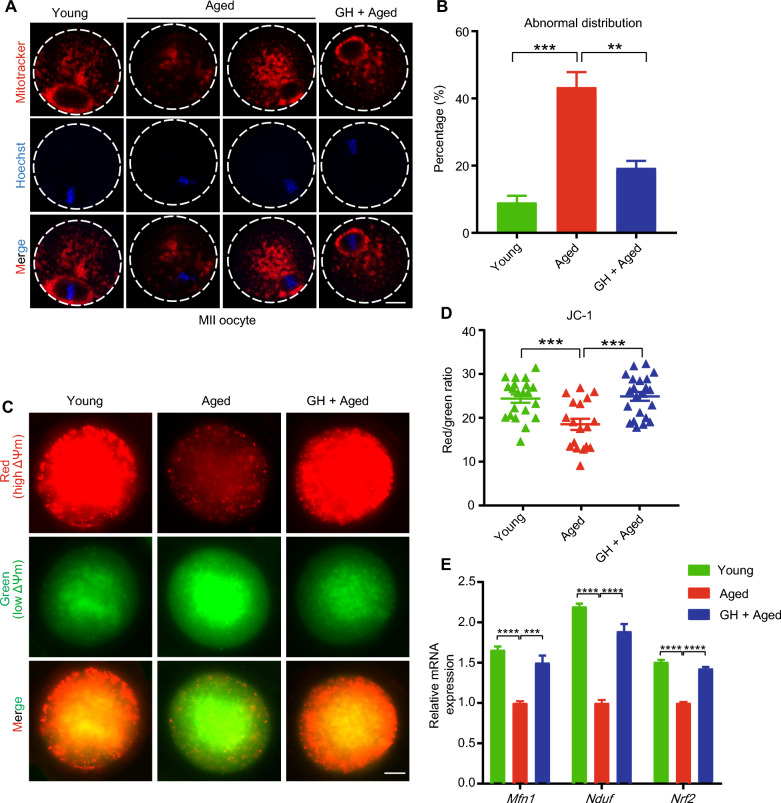
Effect of GH on mitochondrial function in aged oocytes. **A** Representative images of the mitochondrial distribution in young, aged, and GH + aged MII oocytes. Scale bar, 20 μm. **B** The rates of abnormal distribution of mitochondria in young (n = 25), aged (n = 21), and GH + aged (n = 24) oocytes.** C** Representative images of mitochondrial membrane potential (ΔΨm, staining with JC-1) in young, aged, and GH + aged oocytes (red, high ΔΨm; green, low ΔΨm). Scale bar, 20 μm. **D** Red-to-green fluorescence intensity ratio in young (n = 21), aged (n = 18), and GH + aged (n = 22) oocytes. **E** Expression of *Mfn1*, *Nrf2*, and *Nduf* revealed by real-time PCR in young, aged, and GH + aged oocytes. Data are means ± SEM of at least three independent experiments. **p < 0.01, ***p < 0.001, ****p < 0.0001

Mitochondria are important for oocyte maturation and metabolism. The mitochondrial distribution differs between young and aged oocytes [29]. In the young oocytes in this study, mitochondria gathered around the nucleus or were distributed throughout the cytoplasm. In aged oocytes, mitochondria lost their peri-chromosome aggregation and accumulated in the cytoplasm. GH decreased the proportion of mislocalized mitochondria in aged oocytes from 43 to 19% (Fig. 2A and B). We then evaluated the mitochondrial membrane potential (ΔΨm) of oocytes. The results showed that the JC-1 fluorescence intensity (red/green ratio) was weaker in aged than young oocytes, but significantly increased by GH treatment (Fig. 2C and D). Besides, the expression levels of several genes related to mitochondrial fusion (*Mfn1* and *Nduf*) and fission (*Nrf2*) were downregulated in aged oocytes but restored by GH (Fig. 2E).

### GH restored spindle assembly and reduced the rate of aneuploidy in aged oocytes

**Fig. 3:**
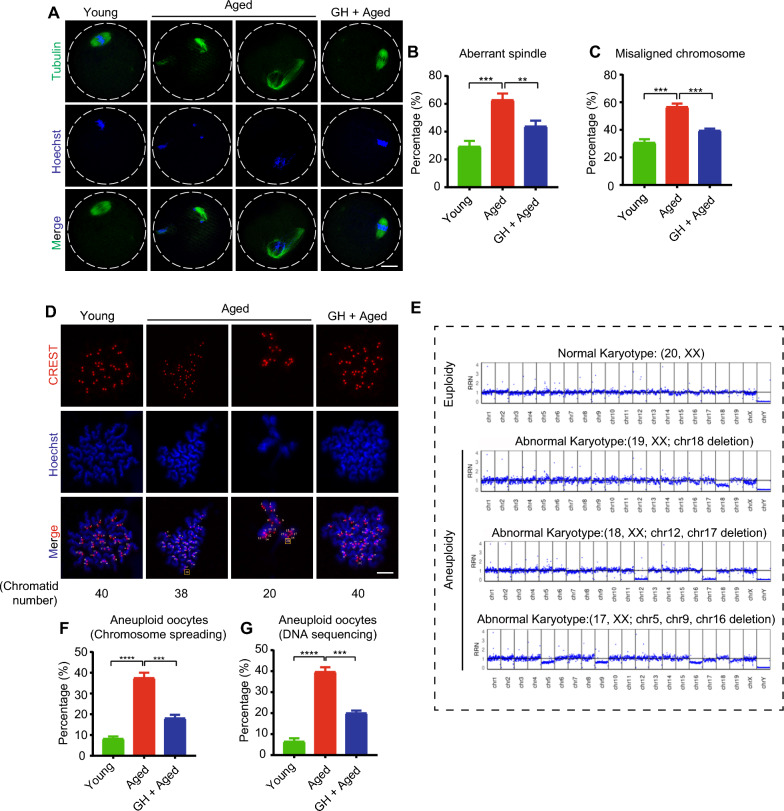
Effect of GH on the spindle/chromosome structure and aneuploidy rate in aged oocytes. **A** Representative images of spindle morphology and chromosome alignment at metaphase II in young, aged, and GH + aged oocytes. Scale bar, 20 μm. **B** and **C** Aberrant spindle and misaligned chromosome rates of young (n = 24), aged (n = 22), and GH + aged (n = 21) oocytes. **D** Representative images of karyotype results obtained from chromosome spreading of MII oocytes from young, aged, and GH + aged groups. Yellow box, the total number of chromatid (normal karyotype is 40 chromatids). Scale bar, 10 μm. **E** Representative images of karyotype results obtained from DNA sequencing of MII oocytes (first polar body removed) from young, aged, and GH + aged groups. The euploid oocytes indicated normal karyotype (with 20 chromosomes), and the aneuploid oocytes showed several abnormal karyotype, including (but not limited to) one (chr18) or more chromosomes (chr12 and chr17; chr5, chr9 and chr16) are missing. **F** Quantification of aneuploidy rate (by chromosome spreading) in young (n = 26), aged (n = 23), and GH + aged (n = 25) oocytes. **G** Aneuploidy rates (by DNA sequencing) of young (n = 26), aged (n = 26), and GH + aged (n = 30) oocytes. Data are means ± SEM of at least three independent experiments. **p < 0.01, ***p < 0.001, ****p < 0.0001

Chromosome movement mistakes are simple to make during oocyte maturation (Additional file 4: Fig. S1). In metaphase II (MII) oocytes, the spindle/chromosome organization was investigated. Young oocytes had a characteristic barrel-shaped spindle with well-aligned chromosomes (Fig. 3A). Aged oocytes had a higher percentage of spindle/chromosome defects, which was greatly decreased by GH (Fig. 3A–C).

Given the relationship between abnormal spindle/chromosome structure and aneuploidy, we performed chromosome spreading to count the chromosome. The typical mouse karyotype has 20 chromosomes and 40 chromatids. As expected, aged oocytes had a higher rate of aneuploidy than young ones, and GH reduced the aneuploidy rate (Fig. 3D and F). Then, we carried out further DNA sequencing to determine the oocytes’ karyotype. Aged oocytes consistently exhibited an increased percentage of aneuploidy, including but not limited to one or more missing chromosome. However, GH reduced the percentage of aneuploidy in aged oocytes from 39.7% to 20.0% (Fig. 3E and G). Therefore, GH decreased nn the rates of spindle/chromosome defects and aneuploidy in aged oocytes.

### GH improved the developmental potential of aged oocytes

**Fig. 4:**
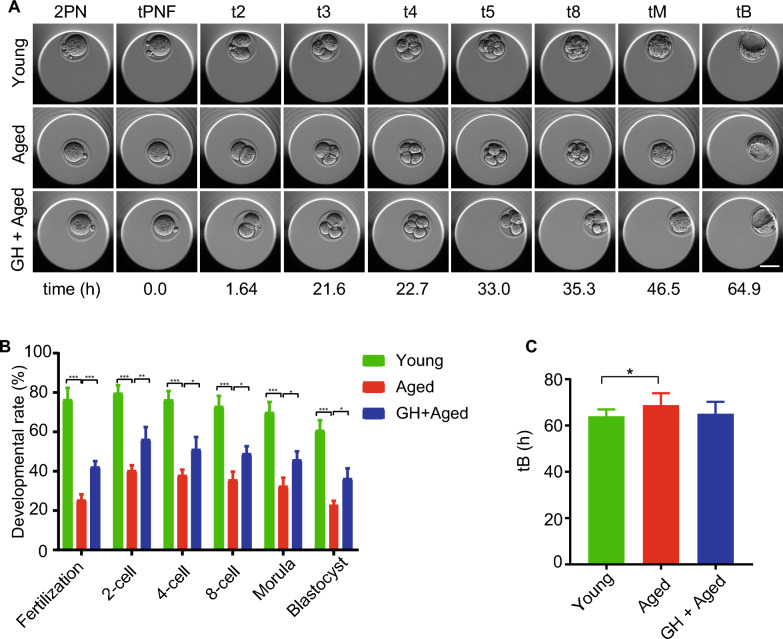
Effect of GH on the development potential of aged oocytes. **A** Representative time-lapse images of young, aged, and GH + aged embryos at the indicated stages. 2PN, two pronuclei; tPNF, time to pronuclei fading; t2, t3, t4, t5, and t8, time in hours post-tPNF for the embryo to reach the 2-, 3-, 4-, 5-, and 8-cell stages, respectively; tM, time for compaction; tB, time for the blastocoel to reach greater than or equal to half the volume of the embryo. Scale bar, 50 μm. **B** Fertilization, 2-, 4-, and 8-cell embryo, morula, and blastocyst rates in young (n = 21), aged (n = 18) and GH + aged (n = 22) groups. **C** Times for embryos to reach the blastocyst stages in the young (n = 21), aged (n = 18), and GH + aged (n = 22) groups. Data are means ± SEM. *p < 0.05, **p < 0.01, ***p < 0.001

In IVF experiments, embryos was put into time-lapse incubator to record developmental potential (Fig. 4A). The results showed that the number of sperm binding to the zona pellucida (Additional file 5: Fig. S2), and the fertilization rate (Fig. 4B), were markedly decreased in aged oocytes but this was rescued by GH. Also, GH improved the rates of two-cell, four-cell, eight-cell, morula, and blastocyst (Fig. 4B). Time-lapse monitoring (Fig. 4A) showed that the interval from zygote to blastocyst was longer in the aged compared to young oocytes (Fig. 4C); GH non-significantly decreased the time to blastocyst formation in aged oocytes (Fig. 4C). Therefore, the data showed that GH improved the fertilization ability and developmental potential of aged oocytes.

### Proteomics analysis of DE proteins in aged oocytes

**Fig. 5:**
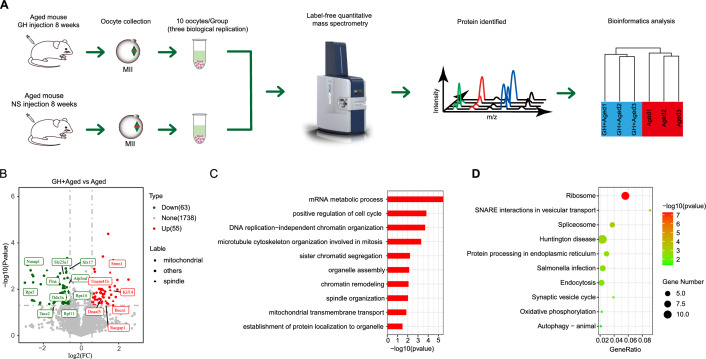
Proteomics and bioinformatics analyses of GH + aged compared to aged MII oocytes. **A** Schematic diagram of the proteomics analysis, and results for GH + aged (blue) and aged (red) MII oocytes (first polar body removed). **B** Volcano plot of the 118 DE proteins (downregulated, green; upregulated, red) in GH + aged compared to aged oocytes. Highly DE proteins are listed, and 16 proteins related to spindle assembly (triangle) and mitochondria (square) are labelled. **C** GO enrichment analysis of the 118 DE proteins in GH + aged compared to aged group, performed using Metascape. **D** KEGG pathway enrichment analysis of the 118 DE proteins in GH + aged compared to aged group, performed using Metascape. The ratio of involved genes to total genes was calculated

Proteomics analysis of MII oocytes yielded a total of 1,856 proteins (Fig. 5A and Additional file 2: Table S2). Unsupervised hierarchical clustering and principal component analysis (PCA) showed high intragroup reliability, and clearly distinguished the GH + aged and aged groups (Fig. 5A). We identified 118 DE proteins in the GH + aged group (p < 0.05, fold change > 1.5) (Fig. 5B, Additional file 2: Table S2 and Additional file 3: Table S3), including 63 upregulated and 55 downregulated proteins.

The GO functional enrichment results are shown in Fig. 5C. Regarding biological processes, some DE proteins were related to “positive regulation of cell cycle”, “DNA replication”, “spindle organization”, “sister chromatid segregation”, and “mitochondrial transmembrane transport.” KEGG pathway analysis showed enrichment of several signaling pathways related to oocyte quality, including “oxidative phosphorylation” and “autophagy” (Fig. 5D). Therefore, most of the enriched biological processes and pathways were consistent with our finding that GH altered mitochondrial function and spindle organization in aged oocytes.

### MAPK3/1 pathway was involved in GH improving the quality of aged oocytes

**Fig. 6:**
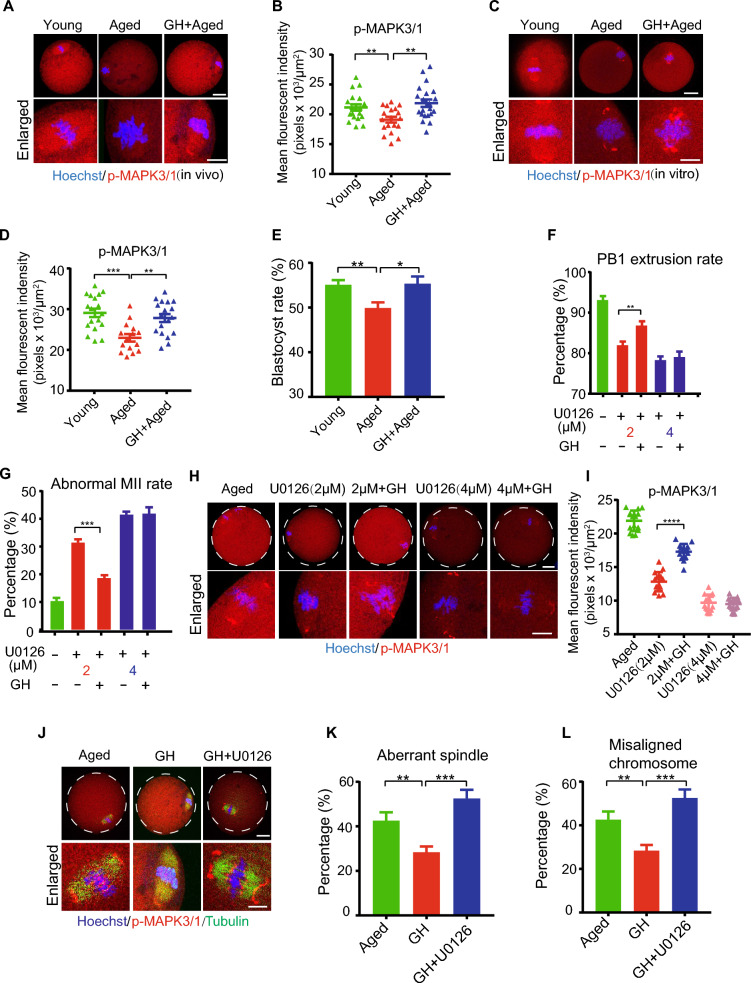
GH improved the quality of aged oocytes through MAPK3/1 pathway. **A** Representative images of p- MAPK3/1 expression in young, aged, and GH + aged oocytes (in vivo). Scale bar, 20 μm. **B** p- MAPK3/1 fluorescence intensity in young (n = 19), aged (n = 19), and GH + aged (n = 21) oocytes. **C** Representative images of p- MAPK3/1 expression in young, aged, and GH + aged oocytes (in vitro). Scale bar, 20 μm. **D** p- MAPK3/1 fluorescence intensity in young (n = 18) and, aged (n = 15), and GH + aged (n = 18) oocytes. **E** Development rate (2-, 4-, and 8-cell embryo, morula and blastocyst stages) of young (n = 31), aged (n = 28) and GH + aged (n = 33) in vitro-matured oocytes.** F** PBE ratio in the presence of 2 and 4 μM MAPK3/1 inhibitor (U0126). **G** Abnormal MII rate in the presence of 2 and 4 μM U0126.** H** Representative images of p- MAPK3/1 expression in aged, U0126 (2 μM), U0126 (2 μM) + GH, U0126 (4 μM) and U0126 (4 μM) + GH oocytes. Scale bar, 20 μm.** I** p- MAPK3/1 fluorescence intensity in aged (n = 15), U0126 (2 μM) (n = 15), U0126 (2 μM) + GH (n = 15), U0126 (4 μM) (n = 15) and U0126 (4 μM) + GH (n = 15) oocytes.** J** Representative images of spindle morphology and chromosome alignment in aged, GH, and GH + U0126 oocytes. Scale bar, 20 μm. **K** and **L** Aberrant spindle and misaligned chromosome rates in aged (n = 18), GH (n = 17), and GH + U0126 (n = 20) oocytes. Aged, oocytes from aged mice administered PBS; GH, oocytes from aged mice administered GH; GH + U0126, oocytes from aged mice administered U0126 and treated with GH. Data are means ± SEM of at least three independent experiments. *P < 0.05, **p < 0.01, ***p < 0.001, ****p < 0.0001

The DE proteins were enriched in several key pathways related to oocyte quality, including spindle organization, cell cycle and mitochondrial function (Fig. 5C). Analysis of the upstream proteins of these DE proteins using the STRING database [30] indicated the enrichment in the MAPK, TNF, and autophagy pathways (Additional file 6: Fig. S3). MAPK3/1 pathway is known to play critical roles in the regulation of microtubule organization and meiotic spindle assembly during oocyte maturation [31, 32]. Thus, we hypothesized that MAPK3/1 pathway is involved in improving the quality of aged oocytes by GH supplementation. Our results showed that the p-MAPK3/1 immunofluorescence intensity was reduced in aged oocytes compared with young oocytes, but were restored by GH (Fig. 6A and B).

In order to investigate the potential mechanism of GH, we further cultured aged GV oocytes with or without GH during in vitro maturation*.* First, we found that the strongest p-MAPK3/1 signal was seen in aged oocytes treated with 100 ng/mL GH (Additional file 7: Fig. S4A and B); therefore, 100 ng/mL GH was used in subsequent experiments. As expected, GH supplementation in vitro restored the decreased level of MAPK3/1 in aged oocytes (Fig. 6C and D), which were consistent with the in vivo results. During oocyte maturation, the PBE rates were comparable among the three groups (Additional file 8: Fig. S5A and B). However, at 8 and 9 h post-GVBD, more aged oocytes extruded first polar body (PB1), indicating the accelerated meiotic progression in aged oocytes (Additional file 8: Fig. S5A, C, and D). In addition, GH supplementation increased the blastocyst formation rate of aged oocytes (Fig. 6E). To verify whether MAPK3/1 is involved in GH effect on improving the maturation of aged oocytes, MAPK3/1 specific inhibitor (U0126) was used. The results showed that GH to some extent restored the PBE rate and the morphology of MII oocytes in the presence of 2 μM, but not 4 μM, U0126 (Fig. 6F and G). Also, the effect of GH on the recovery of MAPK3/1 was represented in 2 μM group, but disappeared in 4 μM group (Fig. 6H and I). Furthermore, we found that normal spindle/chromosome structure was accompanied by more MAPK3/1 localization, and GH reduced the spindle/chromosome defects in aged oocytes. The recovery effects of GH could be counteracted by MAPK3/1 blocking (Fig. 6J–L).

### JAK2 might be involved in the regulation of MAPK3/1 by GH in aged oocytes

**Fig. 7:**
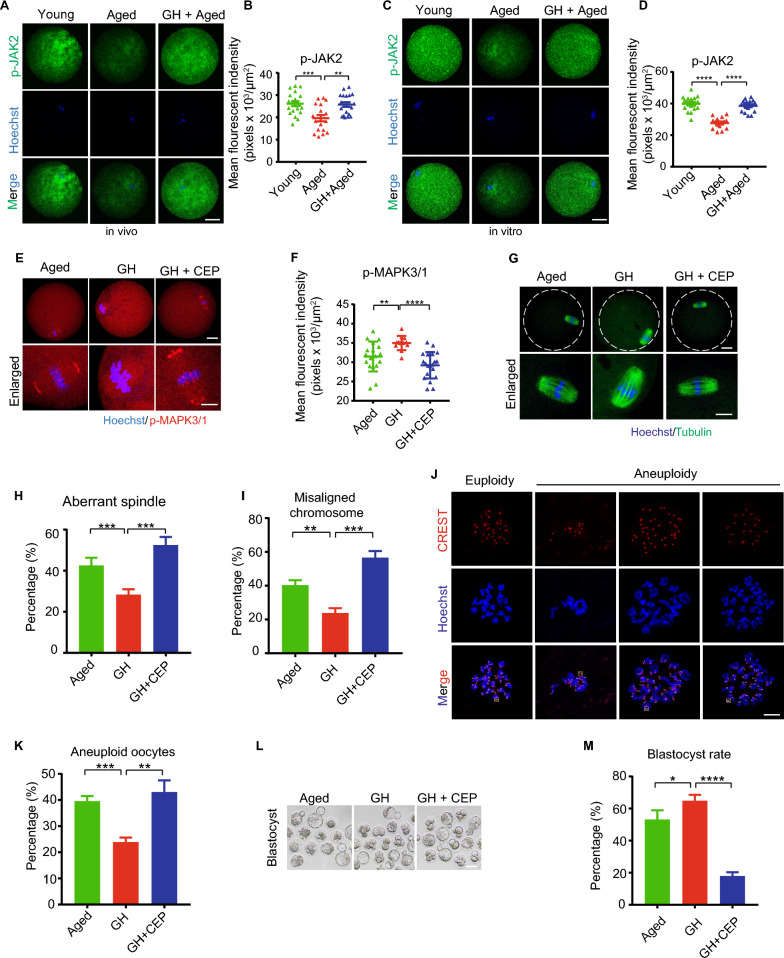
JAK2 might be involved in the regulation of MAPK3/1 by GH in aged oocytes.** A** Representative images of p-JAK2 in young, aged, and GH + aged oocytes (in vivo). Scale bar, 20 μm. **B** p-JAK2 fluorescence intensity in young (n = 20), aged (n = 18), and GH + aged (n = 19) oocytes. **C** Representative images of p-JAK2 in young, aged and GH + aged oocytes (in vitro). Scale bar, 20 μm. **D** The fluorescence intensity of p-JAK2 was measured in young (n = 17), aged (n = 15) and GH + aged (n = 18) oocytes.** E** Representative images of p- MAPK3/1 in aged, GH, and GH + CEP oocytes. Scale bar, 20 μm. **F** p-MAPK3/1 fluorescence intensity in aged (n = 18), GH (n = 12), and GH + CEP (n = 20) oocytes. **G** Representative images of spindle morphology and chromosome alignment in aged, GH, and GH + CEP oocytes. Scale bar, 20 μm. **H** and **I** Aberrant spindle and misaligned chromosome rates of aged (n = 27), GH (n = 22), and GH + CEP (n = 28) oocytes. **J** Representative images of chromosome spreading of euploid and aneuploid oocytes. Scale bar, 10 μm.** K** Aneuploidy rate in aged (n = 25), GH (n = 23), and GH + CEP (n = 26) oocytes. Scale bar, 50 μm. **L** Representative images of early embryos developed from aged, GH, and GH + CEP oocytes. Scale bar, 50 μm. **M** Blastocyst formation rate of aged (n = 25), GH (n = 34), and GH + CEP (n = 8) oocytes. Aged, oocytes from aged mice administered PBS; GH, oocytes from aged mice administered GH; GH + CEP, oocytes from aged mice administered CEP and treated with GH. Data are means ± SEM of at least three independent experiments. *p < 0.05, **p < 0.01, ***p < 0.001

Previous studies have indicated that Janus kinase 2 (JAK2) is one of the Janus kinase family, and plays essential roles in signal transduction of GH [33]. In this study, we found that the level of JAK2 was reduced in aged oocytes compared with young ones, but was restored by GH supplementation in vivo (Fig. 7A and B). Similar results were observed in in vitro study (Fig. 7C and D). Meanwhile, inhibition of JAK2 by CEP neither recover the protein amount of p-MAPK3/1 (Fig. 7E and F), nor restore meiotic defects in aged oocytes (Fig. 7G–K). In addition, the positive effects of GH on developmental potential of aged oocytes could be disrupted by JAK2 blocking (Fig. 7L and M). These data suggested that JAK2 might be involved in the regulation of MAPK3/1 by GH in aged oocytes.

## Discussion

Previous studies have reported that GH treatment improved the IVF outcomes of patients with POR [34, 35], suggesting its possible role in improving the quality of oocytes. In this study, we have confirmed that in vivo administration of GH does improve the oocyte quality in aged mice by reducing the occurrence of aneuploidy. Besides, our proteomics analysis and in vitro studies showed that GH reduced the aneuploidy rate of aged oocytes possibly by JAK2-MAPK3/1 pathway.

To date, the role of GH in ART is still controversial. Some clinical studies have indicated that GH increases the number of oocytes retrieved and subsequent embryo development, as well as the clinical pregnancy and live birth rates, in POR patients [19, 36–38]. However, other studies showed no effect of GH on implantation and clinical pregnancy rates [19, 39, 40]. The low fertility of older women may be related to an aging-related decline in GH level [41]. The GH level is markedly lower in infertile women aging over 30 years but pregnancy outcome is significantly increased by GH supplementation [41]. These data have offered the evidence for possible beneficial effect of GH supplementation in women with advanced age.

In investigations by Hou et al*.* [22] and Feng et al*.* [42], mice were given to low- (0.4), medium- (0.8) and high-doses (1.6 mg/kg/day) of GH, and it was discovered that the high-dose group of mice retrieved more oocytes and had improved mitochondrial function. In our pre-experiments, we used 1.0 and 1.6 mg/kg/day for 8 weeks, and we found that GH at a dose of 1.6 mg/kg/day increased the number of retrieved oocytes. Therefore, we used 1.6 mg/kg/day of GH in the subsequent in vivo studies. Additionally, we tested 12-month-old mice, which already have a high level of aneuploidy, but no discernible effect of GH was seen. In this study, we administered GH to mice during the aging process from 8 to 10 month old, and discovered that GH reduced aneuploidy of oocytes during aging process. Therefore, we would prefer to believe that GH supplementation is to prevent the defects to occur in aged oocytes.

In this study, we found that GH enhanced mitochondrial function by restoring the mitochondrial distribution and elevating the mitochondrial membrane potential (ΔΨm) in aged oocytes. It is reported that mitochondrial functions, including ATP production, lipid biogenesis and protein transport depend on the maintenance of ΔΨm; and sufficient ATP generation ensures the availability of energy for oocyte meiotic progression and early embryonic development [43–45]. Consequently, GH-mediated mitochondrial function enhancement increased of aged oocyte quality and developmental potential.

To date, the potential mechanism of GH on oocyte quality improvement is still unknown. To ascertain the mechanisms of how GH improved the quality of aged oocytes, we performed proteomics analysis to identify the potential target effectors. Our data showed that the DE proteins were involved in spindle organization and mitochondrial function. Then, we found that the upstream genes related to DE proteins were enriched in the MAPK pathway, of which MAPK3/1 are the best-characterized. Yonatan B e*t al.* have reported that MAPK signaling regulates meiosis progression and oocyte aging [46–48]. In mammalian oocytes, MAPK3/1 activity is crucial for first meiosis progression and MII arrest maintenance [49–51]. MAPK3/1 regulates microtubule organization and spindle assembly during oocyte meiotic maturation [31, 32]. In this study, we found that the PBE took place earlier in the aged oocytes, indicating an accelerated first meiosis in aged oocytes. Spindle assembly checkpoint (SAC) is used in oocytes to prevent premature chromosome segregation during meiosis. When chromosomes are correctly aligned at the metaphase plate, SAC activity is silenced and promote cell cycle entry into anaphase. Angell et al*.* [52] have demonstrated that, compared with young oocytes, SAC proteins were less in aged oocytes, which would enter the anaphase of cell cycle early and aggravate the meiosis in aged oocytes. In the present study, we found that the level of p- MAPK3/1 was lower in aged oocytes than that in young ones, and the aneuploidy rate was increased in aged oocytes. These observations implied MAPK3/1 was linked to aneuploidy in aged oocytes. In addition, our results showed that GH supplementation increased p- MAPK3/1, and decreased the aneuploidy rate in aged oocytes. Also, inhibition of MAPK3/1 resulted in higher percentage of spindle/chromosome defects, which was to some extent restored by GH, thereby increasing the developmental potential of aged oocytes. Therefore, our data indicated MAPK3/1 pathway might be involved in GH-mediated reduction of the aneuploidy in aged oocytes.

The first step in GH action is its binding to the GHR, and this signaling partly depends on the level of GHR at the cell surface. Here, we found that the GHR level of aged oocytes was increased by GH supplementation. Several studies have demonstrated that in preadipocytes and endometrial adenocarcinoma cells, the GHR can activate JAK2 and then promote the MAPK pathway [53, 54]. However, whether oocytes possess the same molecular regulation is still unknown. In mouse oocytes and early cleavage stage embryos, JAK2 locates to the chromosomes, and regulates the microfilaments aggregation during oocyte maturation [55]. In this study, our data confirmed that increased p-JAK2 expression in the GH-supplemented oocytes enhanced p-MAPK3/1 level, thus leading to the reduction of aneuploidy and improvement of oocyte quality. However, these observations could be disrupted by the inhibition of JAK2 activity, which indicated that JAK2-MAPK3/1 pathway might mediated the amelioration of aged oocytes quality by GH.

The findings demonstrated that GH supplementation could increase the number of oocytes and reduce the aneuploidy of aged oocytes. Our data indicated that GH decreased aneuploidy possibly by JAK2- MAPK3/1 pathway, thereby improving the quality of aged oocytes. These results provide insight into the mechanism by which GH improves reproductive physiology. Further work is needed to determine whether the GH effects and related mechanisms are conserved between mouse and human to facilitate clinical application of GH.

## Supplementary Information

**Additional file 1: Table S1.** Primer sequences of genes for quantitative real-time PCR.

**Additional file 2: Table S2.** The total proteins detected by proteomics analysis.

**Additional file 3: Table S3.** The DE proteins detected by proteomics analysis.

**Additional file 4: Figure S1.** Meiotic dynamics of chromosomes in mouse oocytes. Oocytes were microinjected with H2B-cherry mRNA, maintained for 2 h in milrinoneand washed with milrinone-free medium to allow development to the GVBD, M I, AT I, and M II stages. PB, polar body. Scale bar, 30 μm.

**Additional file 5: Figure S2.** Effect of GH on the fertilization ability of aged oocytes. Representative images of sperm binding to the zona pellucida of young, aged, and GH + aged oocytes. Scale bar, 20 μm. Number of sperm binding to the surface of the zona pellucida surrounding young, agedand GH + agedoocytes. Data are means ± SEM of at least three independent experiments. **p < 0.01.

**Additional file 6: Figure S3.** Analysis of predicted associated genesfor DE proteins. The PAG for DE proteins were analyzed using STRING, followed by KEGG pathway analysis using DAVID. A. Network of DE proteins and its related PAG. B. The bar chart of KEGG pathway.

**Additional file 7: Figure S4.** Effect of GH on MAPK3/1 pathway in aged oocytes in vitro. Representative images of p- MAPK3/1 in the presence of 100, 200, 400, and 800 ng/mL GH. Scale bar, 20 μm. p- MAPK3/1 fluorescence intensity in the presence of aged, 100 ng/mL, 200 ng/mL, 400 ng/mLand 800 ng/mL GH. Data are means ± SEM of at least three independent experiments. ****p < 0.0001.

**Additional file 8: Figure S5.** Effect of GH on the meiotic progression of aged oocytes in vitro.Representative time-lapse images of PBE kinetics in young, aged and GH + aged oocytes. Scale bar, 50 μm. PBE rate in young, aged, and GH + agedoocytes in vitro PBE kinetics in young, aged, and GH + agedoocytes post-GVBD. Interval from GVBD to PBE in young, aged, and GH + agedoocytes. Data are mean ± SEMs of at least three independent experiments. *p < 0.05.

## Data Availability

The original proteomics data has been uploaded to ProteomeXchange (Project accession: PXD039147). The readers are accessible to the data once the first publication of the manuscript.

## Abbreviations

ART: Assisted reproductive technology
COCs: Cumulus-oocyte complexes
IVF: In vitro fertilization
POR: Poor ovarian response
GV: Germinal vesicle
JAK2: Janus kinase 2
hCG: Human chorionic gonadotropin
NS: Normal saline
ΔΨm: Membrane potential

## Declarations

### Content for publication

The manuscript does not contain any form of personal data.
