# Supplementary material for: Growth hormone reduces aneuploidy and improves oocytes quality by JAK2-MAPK3/1 pathway in aged mice
Source: J Transl Med. 2023 Jun 29;21:426. doi: 10.1186/s12967-023-04296-z (PMC10311773; doi:10.1186/s12967-023-04296-z)
Supplement: Supplementary file 1 — Additional file 1: Table S1. Primer sequences of genes for quantitative real-time PCR. [file 12967_2023_4296_MOESM1_ESM.docx]

**Table S1. Primer sequences of genes for quantitative real-time PCR**

| Gene | Primer sequence |
| --- | --- |
| *GHR* | F: CTGCAAAGAATCAATCCAAGCC |
|  | R: CAGTTCAGGGGAACGACACTT |
| *Mfn1* | F: GGACTTTATCCGAAACCA GA |
|  | R: TGAGATTGAAGAATGGAGGC |
| *Nrf2* | F: GGTCACGCTAATGCAGACAAT |
|  | R: TCTTCTCAGGGGTATTCGCTTT |
| *Nduf* | F: GAGGTTGCTGAGACTCGTCC |
|  | R: CCATCTACTGTTATCACTCGGCT |
