# Supplementary figures and images for: Growth hormone reduces aneuploidy and improves oocytes quality by JAK2-MAPK3/1 pathway in aged mice
Source: J Transl Med. 2023 Jun 29;21:426. doi: 10.1186/s12967-023-04296-z (PMC10311773; doi:10.1186/s12967-023-04296-z)

## Normal

## Abnormal

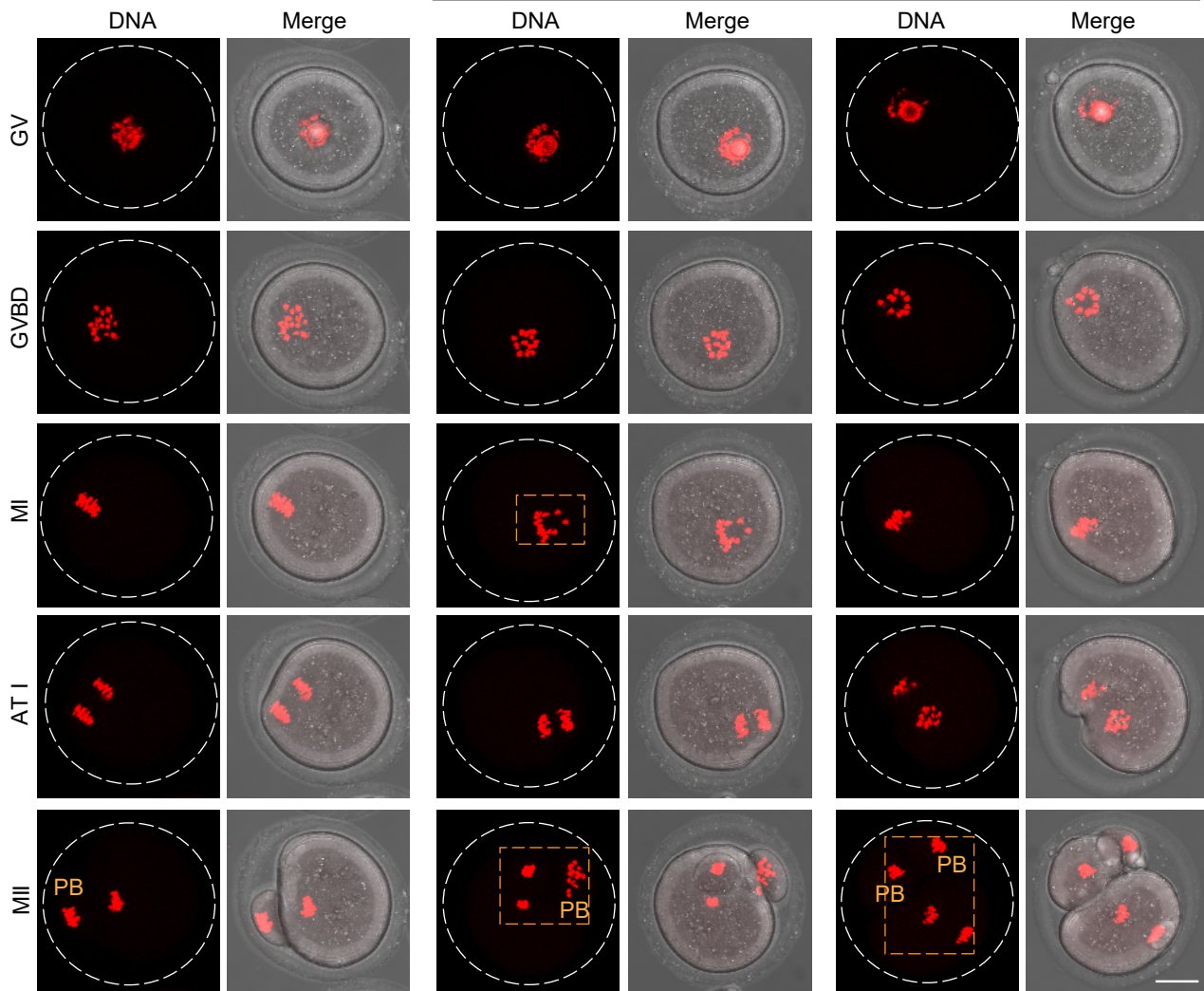

Supplement: Supplementary file 4 — Additional file 4: Figure S1. Meiotic dynamics of chromosomes in mouse oocytes. Oocytes were microinjected with H2B-cherry mRNA, maintained for 2 h in milrinoneand washed with milrinone-free medium to allow development to the GVBD, M I, AT I, and M II stages. PB, polar body. Scale bar, 30 μm. [file 12967_2023_4296_MOESM4_ESM.pdf]

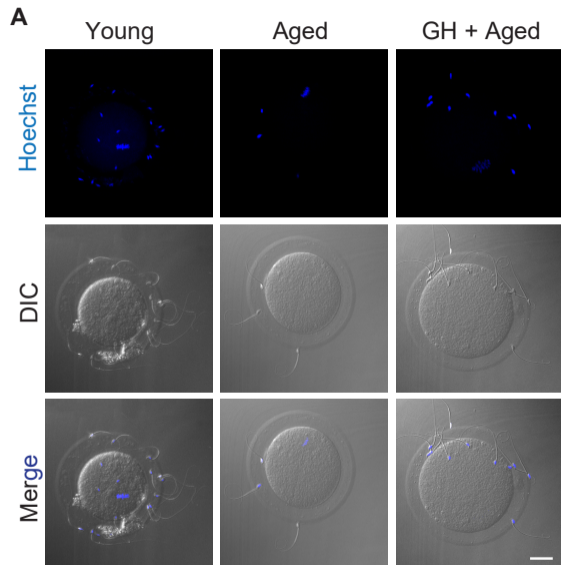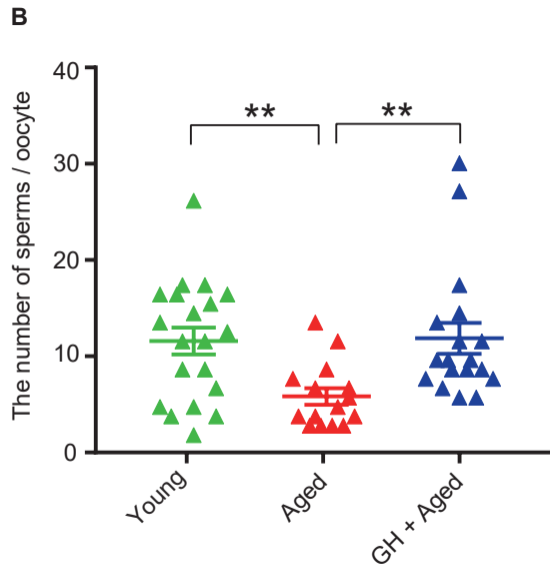

Supplement: Supplementary file 5 — Additional file 5: Figure S2. Effect of GH on the fertilization ability of aged oocytes. Representative images of sperm binding to the zona pellucida of young, aged, and GH + aged oocytes. Scale bar, 20 μm. Number of sperm binding to the surface of the zona pellucida surrounding young, agedand GH + agedoocytes. Data are means ± SEM of at least three independent experiments. **p < 0.01. [file 12967_2023_4296_MOESM5_ESM.pdf]

**A**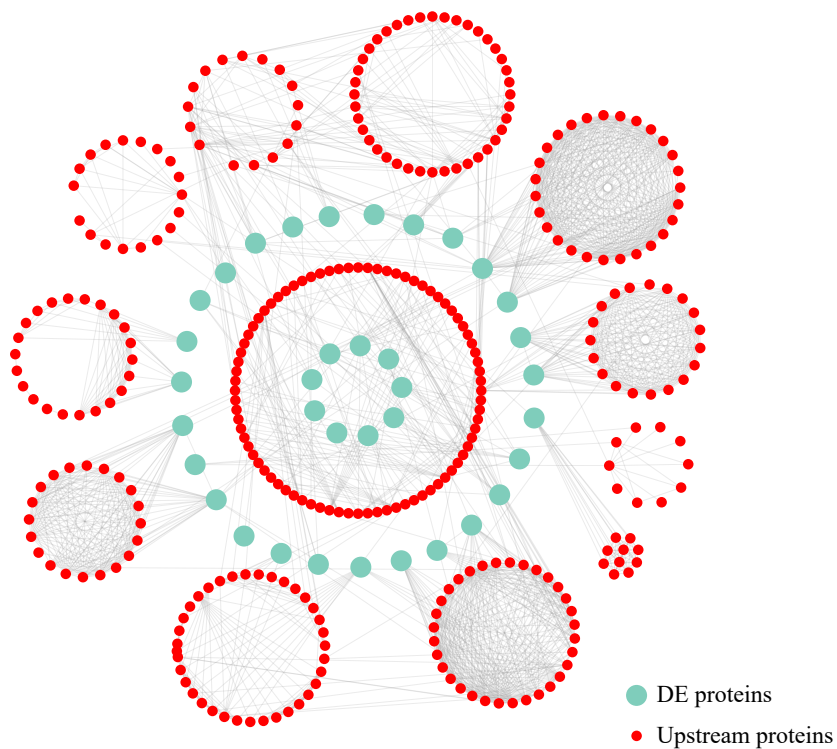**B**

### KEGG pathway

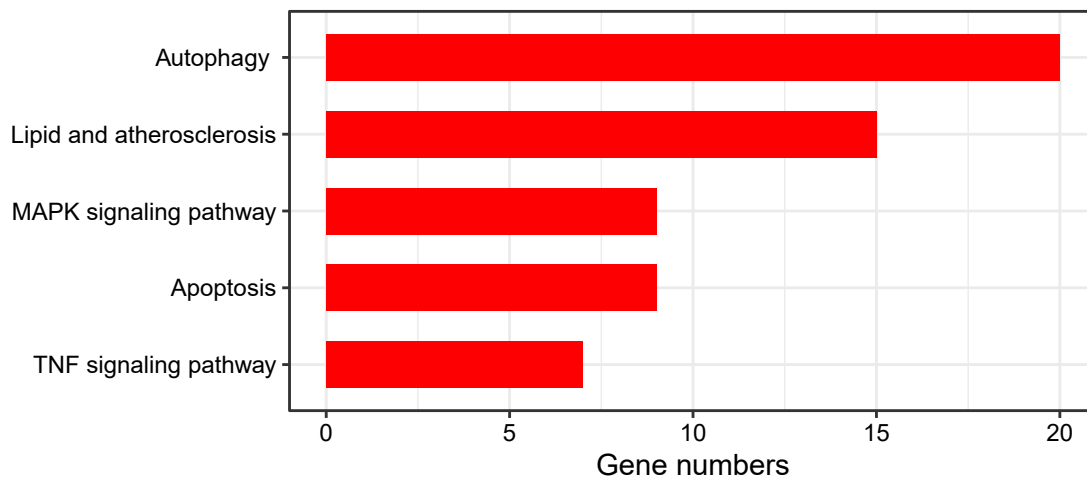

Supplement: Supplementary file 6 — Additional file 6: Figure S3. Analysis of predicted associated genesfor DE proteins. The PAG for DE proteins were analyzed using STRING, followed by KEGG pathway analysis using DAVID. A. Network of DE proteins and its related PAG. B. The bar chart of KEGG pathway. [file 12967_2023_4296_MOESM6_ESM.pdf]

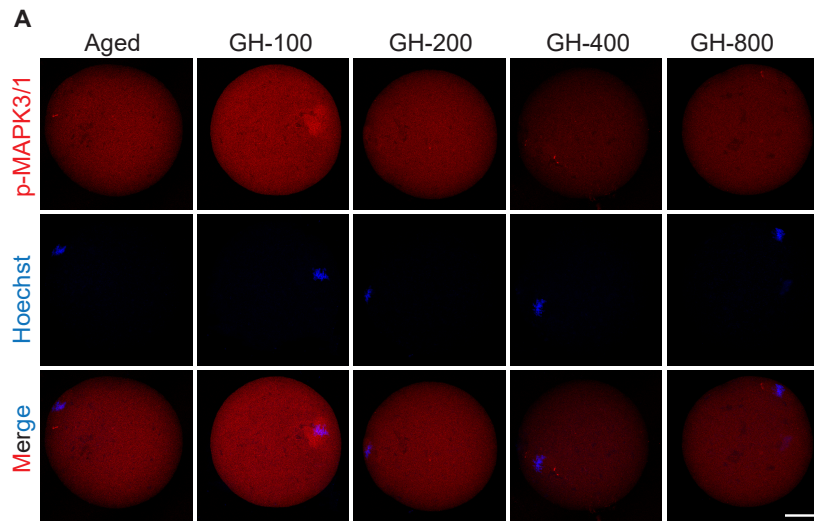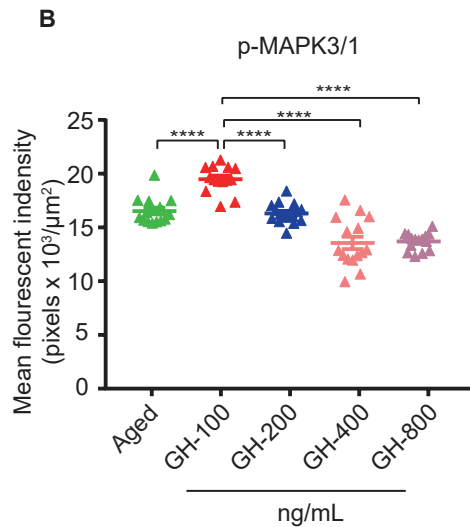

Supplement: Supplementary file 7 — Additional file 7: Figure S4. Effect of GH on MAPK3/1 pathway in aged oocytes in vitro. Representative images of p- MAPK3/1 in the presence of 100, 200, 400, and 800 ng/mL GH. Scale bar, 20 μm. p- MAPK3/1 fluorescence intensity in the presence of aged, 100 ng/mL, 200 ng/mL, 400 ng/mLand 800 ng/mL GH. Data are means ± SEM of at least three independent experiments. ****p < 0.0001. [file 12967_2023_4296_MOESM7_ESM.pdf]

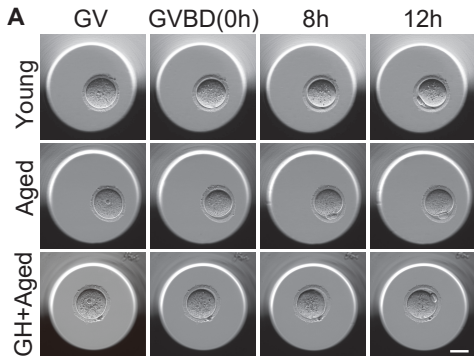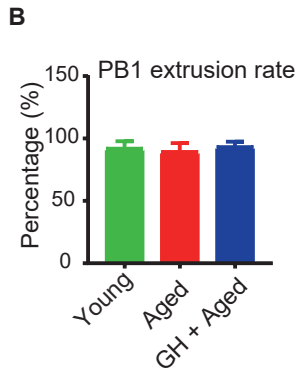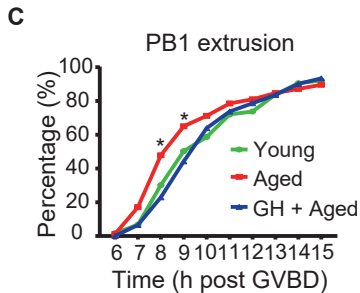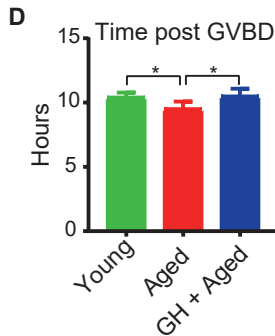

Supplement: Supplementary file 8 — Additional file 8: Figure S5. Effect of GH on the meiotic progression of aged oocytes in vitro.Representative time-lapse images of PBE kinetics in young, aged and GH + aged oocytes. Scale bar, 50 μm. PBE rate in young, aged, and GH + agedoocytes in vitro PBE kinetics in young, aged, and GH + agedoocytes post-GVBD. Interval from GVBD to PBE in young, aged, and GH + agedoocytes. Data are mean ± SEMs of at least three independent experiments. *p < 0.05. [file 12967_2023_4296_MOESM8_ESM.pdf]
